# Enabling Secure Communication in Wireless Body Area Networks with Heterogeneous Authentication Scheme

**DOI:** 10.3390/s23031121

**Published:** 2023-01-18

**Authors:** Insaf Ullah, Muhammad Asghar Khan, Ako Muhammad Abdullah, Fazal Noor, Nisreen Innab, Chien-Ming Chen

**Affiliations:** 1 Hamdard Institute of Engineering & Technology, Islamabad 44000, Pakistan; 2 Computer Science Department, College of Basic Education, University of Sulaimani, Sulaimaniyah 00964, Kurdistan Region, Iraq; 3 Department of Information Technology, University College of Goizha, Sulaimaniyah 00964, Kurdistan Region, Iraq; 4 Faculty of Computer and Information Systems, Islamic University of Madinah, Madinah 400411, Saudi Arabia; 5 Department of Computer Science and Information Systems, College of Applied Sciences, AlMaarefa University, P.O. Box 71666, Riyadh 11597, Saudi Arabia; 6 College of Computer Science and Technology, Shandong University of Science and Technology, Qingdao 266590, China

**Keywords:** WBANs, Fifth Generation (5G), Hyperelliptic Curve Cryptography (HECC), heterogeneous cryptography, authentications

## Abstract

Thanks to the widespread availability of Fifth Generation (5G) wireless connectivity, it is now possible to provide preventative or proactive healthcare services from any location and at any time. As a result of this technological improvement, Wireless Body Area Networks (WBANs) have emerged as a new study of research in the field of healthcare in recent years. WBANs, on the one hand, intend to gather and monitor data from the human body and its surroundings; on the other hand, biomedical devices and sensors interact through an open wireless channel, making them exposed to a range of cyber threats. However, WBANs are a heterogeneous-based system; heterogeneous cryptography is necessary, in which the transmitter and receiver can employ different types of public key cryptography. This article proposes an improved and efficient heterogeneous authentication scheme with a conditional privacy-preserving strategy that provides secure communication in WBANs. In the proposed scheme, we employed certificateless cryptography on the client side and Identity-Based Cryptography on the receiver side. The proposed scheme employs Hyperelliptic Curve Cryptography (HECC), a more advanced variation of Elliptic Curve Cryptography (ECC). HECC achieves the same level of security with a smaller key size and a more efficient approach than its counterpart methods. The proposed scheme not only meets the security and privacy standards of WBANs but also enhances efficiency in terms of computation and communication costs, according to the findings of the security and performance analysis.

## 1. Introduction

**Figure 1:**
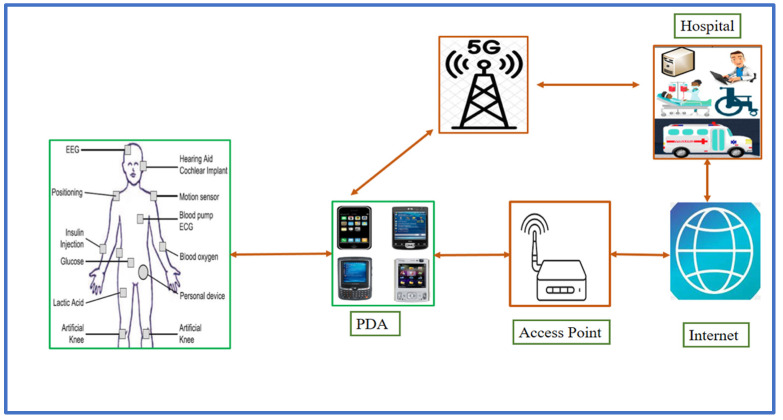
Sample Architecture of WBANs.

WBANs (Wireless Body Area Networks) are a collection of medical devices and software applications that collect, analyze, and communicate the physiological data of patients [1,2]. WBANs have recently received more attention as a result of recent technological breakthroughs in the fields of electronics, sensors, and wireless communication technologies. Due to the wide spread availability of 5G wireless technology, patients can now obtain preventative or proactive healthcare treatments from any location and at any time. Blood pressure, heart rate, body temperature, respiratory rate, electrocardiogram, patient posture, breathing rate, and other signals can all be gathered, analyzed, and shared in real time between both the patient’s own electronic devices and the medical practitioner [3,4,5,6,7]. WBANs can also provide information on patient care settings, room conditions, laboratory shift timings, treatment durations, and staff-to-patient ratios. This information can be saved as an electronic health record in the health information system, which will be accessible to medical experts with a single click whenever the patient visits the hospital. Figure 1 depicts the general architecture of WBANs, in which sensor nodes gather and transfer real-time physiological data from patients to an AP and a typical smart medical service.

Because a considerable number of interactions between biomedical sensors and devices occur via the Internet, security and privacy concerns over sensitive patient data have arisen in WBANs [8]. An intruder, for example, may intercept a communication connection between biomedical devices and sensors in order to steal or manipulate patient health data. As a result, authentication mechanisms are essential to ensure secure communication in WBANs, as well as the privacy of patients’ health-related information. Unfortunately, since most WBANs devices have limited processing and storage capacity, they are unable to execute traditional authentication mechanisms that require complex cryptographic computations, rendering them ineffective for WBANs. As a result, most public key cryptosystems published in the literature require a large number of computations, making them unsuitable for WBAN implementation.

Authentication in cryptography is accomplished by the digital signature procedure, which can be utilized for secure communication in WBANs [9,10]. A shared key is typically used to secure not just authentication and privacy but also confidentiality, integrity, and non-repudiation [11,12]. Identity-Based Cryptography (IBC) and Public Key Infrastructure (PKI) are the two most used ways for validating public keys in public-key cryptosystems. The CA specifies the public keys with the certificates as a participant. On the other hand, PKI systems include downsides such as certificate lifetimes, distribution, and storage concerns. IBC is instead promoted as a means to lower the expense of managing public keys [13]. When it comes to the cost of private key escrow issues, the trustworthy Private Key Generator (PKG) has firsthand knowledge of the participants’ private keys [14,15]. Finally, the key escrow issue in authentication schemes can be addressed by combining a certificateless cryptosystem with a signature strategy. 

**Figure 2:**
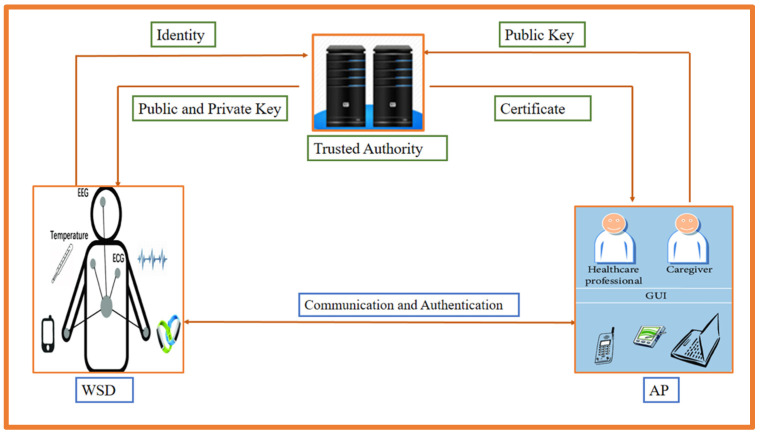
Heterogeneous cryptography (IBC to PKI or PKI to IBC).

**Figure 3:**
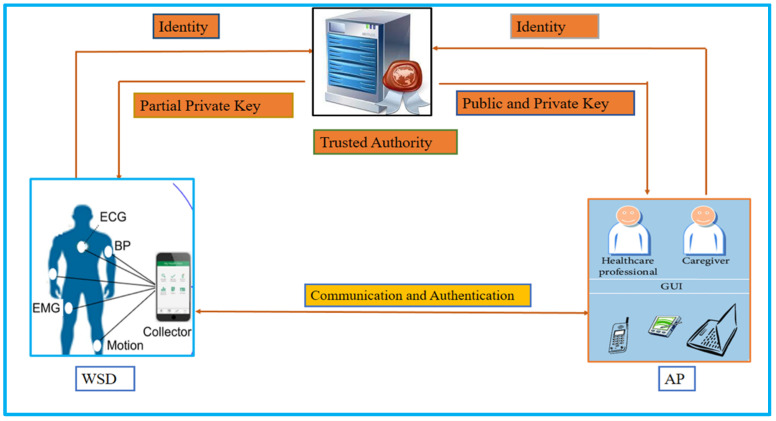
Heterogeneous cryptography (certificateless to IBC).

Although public key cryptosystems are suited for a homogeneous environment, WBANs are a heterogeneous-based system; hence, heterogeneous cryptography is required. The transmitter and receiver in heterogeneous cryptography may use various forms of public key cryptography. In some cases, for example, the sender belongs to IBC, and the receivers use PKI, or the sender uses PKI, and the receivers use IBC. Furthermore, it is possible that the sender uses a certificateless cryptosystem and the receivers use IBC or that the sender uses a certificateless cryptosystem and the receivers use PKI. As a result, in the following Figure 2 and Figure 3, we show the functioning capacity of each of these cryptosystems one by one. Figure 2 shows how we give IBC to the PKI cryptosystem, which includes a Wearable Sensor Device (WSD) injected into the patient’s body, a Trusted Authority (TA), and Application Providers (AP). The process starts when WSD communicate their identities to TA, who then generates the public and private keys for WSD and sends them via a secure network. Following this, WSD may construct the authentication message and transmit it to AP; AP will then give their public key to TA, who will then generate a certificate based on that public key and publicly proclaim it.

In addition, if we regard AP as a transmitter and WSD as a receiver in Figure 2, the PKI to IBC heterogeneous cryptosystem will be represented. Furthermore, we depict the certificateless cryptosystem to IBC in Figure 3, where WSD belongs to certificateless cryptography, and AP uses IBC. TA will produce a partial private key for WSD and transmit it through a secure channel after receiving identification from WSD and AP. TA will also generate a private key for AP and send it via a private network after receiving identity from WSD and AP. After that, the WSD and the AP may communicate and authenticate with each other.

**Figure 4:**
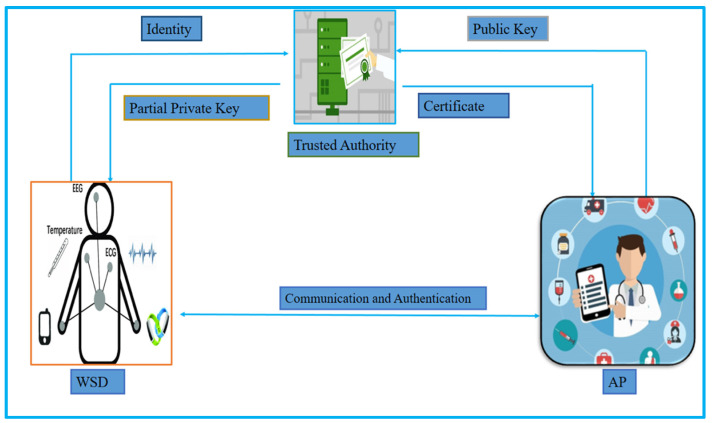
Heterogeneous cryptography (certificateless to PKI).

Figure 4 depicts certificateless to PKI cryptography, with WSD belonging to certificateless cryptography and AP using PKI. TA will construct the partial private key for WSD and transmit it via a secure channel after receiving the identification from WSD and the public key from AP. TA will also create the certificate for AP and send it over to a public network. After that, the WSD and the AP may communicate and authenticate with each other.

In this article, we propose an authentication scheme in heterogeneous settings (certificateless to IBC) based on the discussion above. We considered Hyperelliptic Curve Cryptography (HECC) to create the proposed scheme, which uses just 80-bit keys to give the same level of security in preventing cyber-attacks [16]. As a result, for WBAN devices with limited resources, HECC would be a better option. The following are some of the key contributions of the undertaken research work:

1. We propose a heterogeneous authentication scheme for WBANs that uses the HECC approach, which makes our scheme computationally efficient.
2. Informal security analysis has been used to evaluate the proposed scheme’s ability to withstand different attacks. The results support the proposed scheme’s resiliency.
3. Finally, in terms of computation and communication costs, we compare the proposed scheme to existing equivalent schemes. The result demonstrates that our approach surpasses its competitors.

### Structure of the Paper

The following is how the rest of the article is organized. The related work is detailed in Section 2. The network model is provided in Section 3, followed by the proposed scheme in Section 4. Section 5 and Section 6 contains a security analysis. Section 7 provides a performance evaluation with existing approaches. Concluding remarks are provided in Section 8.

## 2. Related Work

This section covers the existing solutions that have been used to overcome the security and privacy challenges of WBANs that use authentication mechanisms. In 2014, Chen et al. [16] proposed an authentication scheme for medical data exchange in the cloud environment to secure patients’ health information. According to Chiou et al. [17], the approach developed by Chen et al. [16] could not ensure patient confidentiality or message authentication. In [17], the authors improved the privacy authentication process in the cloud health environment.

In 2016, Li et al. [18] introduced a network-based electronic medical authentication scheme that includes two-factor authentication using the user’s password and smart card. He et al. [19] proposed an authentication scheme that is better suited to the setup of telemedicine information systems on mobile devices with minimal battery consumption. Wei et al. [20] observed that this protocol is vulnerable to password attacks; they proposed an improved authentication protocol for telemedicine information systems and showed that it fits the security criteria of two-factor authentication. Wu et al. [21] introduced a lightweight two-factor medical authentication approach in 2018, claiming that their protocol is secure; however, after further investigation, it was shown that their protocol could not successfully resist perfect forward security.

In 2016, Wu et al. [22] proposed a novel anonymous authentication scheme for WBANs and demonstrated that it is secure in a random oracle model. The proposed scheme, on the other hand, was based on bilinear pairing, which entails computationally intensive operations. He et al. [23] proposed a provable security anonymous authentication scheme for WBAN. The proposed scheme [23], on the other hand, comprises a bilinear pairing-based operation, which is a computationally expensive operation. In 2018, Ji et al. [24] proposed a certificateless conditional privacy-preserving authentication technique for WBAN in a big data environment. The proposed technique allows for batch authentication of multiple clients, considerably reducing the service provider’s computing overhead. The proposed scheme supports common security aspects such as user anonymity, unlinkability, mutual authentication, traceability, and forward secrecy. On the basis of assessing the most recently presented certificateless authentication scheme for WBANs, Xie et al. [25] proposed an improved and efficient certificateless authentication scheme with conditional privacy-preserving. However, the proposed scheme was based on elliptic curve cryptography, which is not that suitable for WBAN devices.

Liao et al. [26] proposed a certificateless authentication scheme for WBAN, in which they used the concept of online and offline signature methods. However, the proposed scheme failed to provide real-time communication due to the use of bilinear pairing that needs extra machine time and bandwidth space.

Recently, Li et al. [27] proposed a certificateless authentication with the help of an elliptic curve; however, the proposed scheme failed to provide real-time communication due to the use of an elliptic curve that needs extra machine time and bandwidth space.

The schemes outlined above rely on cryptographic techniques such as ECC and bilinear pairing and have high computation and communication costs. On the other hand, the proposed scheme is based on the concept of HECC, which is a more refined variant of ECC. It provides the same amount of security as other methods but with a smaller key size.

## 3. Network Model

**Figure 5:**
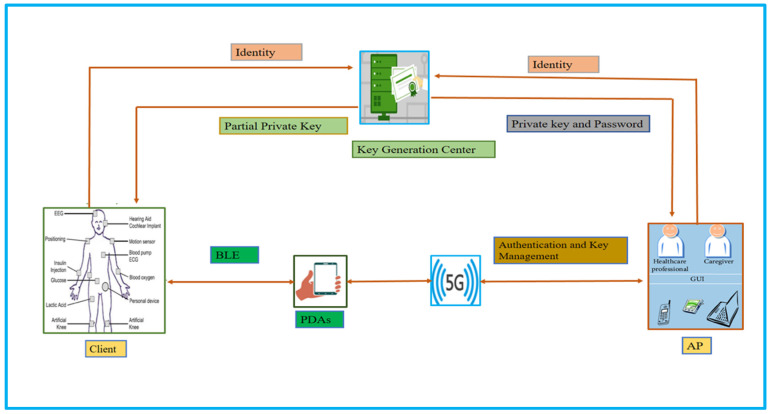
Proposed heterogeneous authentication and key management scheme.

Figure 5 depicts the proposed network’s working flow, in which we considered three main entities that are client, Application Provider (AP), and Key Generation Center (KGC), respectively. The role of each entity is explained as follows.

### 3.1. Client

The client is the sensors placed in the human body, and the work of these sensors is to collect health-related data from the human body. The client sends a request along with their identity for the partial private key to KGC, then by using a secure channel, KGC sends a partial private key to the client.

Further, the collected data, along with a partial private key, is sent by the client through Bluetooth Low Energy (BLE) to PDAs. With the help of the client, PDAs first generate a signature, secret key, public parameter, cipher text, and hash value. Then PDAs will send the hash value, public parameter, ciphertext, and signature to AP through 5G technology.

### 3.2. Application Provider (AP)

This entity sends a request along with its identity to KGC, then the KGC generates and sends a private key to AP through a secure channel. Therefore, upon receiving the hash value, public parameter, ciphertext, and signature, AP first verifies the signature, recovers the secret key, and uses the secret key to recover a message from the ciphertext.

### 3.3. Key Generation Center (KGC)

This entity is responsible for generating the partial private key for the client and the private key for AP.

## 4. Proposed Conditional Privacy-Preserving Authentication Scheme for WBAN

In this section, we first provide Table 1, which includes acronyms used in the article and symbols utilized in the new algorithm. The five stages of our proposed conditional privacy-preserving authentication scheme for WBAN are described [24]:

### 4.1. Setup

The KGC performs the following sub initializations:

It chooses a hyper elliptic curve of genus 2 with 80 bits parameter size;

It also chooses the hash functions, i.e., ($H_{a}{}^{1},H_{a}{}^{2},H_{a}{}^{3},H_{a}{}^{4}$), and its capability as it is irreversible;

Then, it selects 𝓀 randomly from the finite group of hyper elliptic curve and computes α=𝓀.D and set α as the master public key and 𝓀 as the secret key;

Exempt α, the KGC published all the above-discussed parameters in a network;

For AP, it selects φ,Ʈ randomly from the finite group of hyper elliptic curve, calculates ƞ=φ .D, Y=Ʈ .D and sets (ƞ,Y) as the public key and (φ,Ʈ) as the secret key of AP.

### 4.2. Pseudo Identity Generation

A client can select σ randomly from the finite group of hyper elliptic curve and compute S=σ.D, and by using a secure network, it sends ($S,\;Client_{RID},Client_{PW}$) to the KGC, where $Client_{RID}$ is the identity of the client, and $Client_{PW}$ denotes the password of the client. Upon reception ($S,\;Client_{RID},Client_{PW}$), the KGC can select θ randomly from the finite group of hyper elliptic curve and compute ƕ=θ.D, $ℰ=H_{a}{}^{1}\left(Client_{RID}\right)⊕H_{a}{}^{1}\left(Client_{PW}\right)$, $\ell =H_{a}{}^{2}\left(𝓀.S,T_{limit},ƕ\right)$, $Client_{PID}=Client_{RID}⊕\ell$, $J=H_{a}{}^{3}\left(Client_{PID},S,ƕ,T_{limit}\right)$, and Ω=θ+𝓀.J, respectively. Then, KGC saves ($Client_{PID},S,ƕ,T_{limit},\Omega ,ℰ$) in the memory of the controller. Finally, the client can set (Ω,σ) as their private key and (S,ƕ) as their public key.

### 4.3. Mutual Authentication and Secrete Key Management

A client can select χ randomly from the finite group of hyper elliptic curve and compute Q=χ.D, K=χ.ƞ, r= $H_{a}{}^{3}\left(\;Q.S,T_{limit},ƕ,Client_{PID}\right)$, S=φ+Ʈ+r.χ, and send (Q,r, S) to AP.

When AP receives the triple (Q,r, S) then it performs the following step for the verification of the signature received from the client and generation of the secret key.

It computes S.D=Y+ƞ+r.Q if it is qualified, then the client mutually authenticates with AP.

Then AP generates the secret key as K=Q.φ and when it receives an encrypted message as $C=E_{K}\left(medical\;data\right)$ from the client, it performs the decryption process on the same secret key.

### 4.4. Password Change Phase

This phase is the same as the password change process in [1].

### 4.5. Correctness

Here, AP can generate the secret key and verify the signature as follows:

K=Q.φ=Q.φ=χ.D.φ=χ.ƞ, hence proved;

S.D=Y+ƞ+r.Q=(φ+Ʈ+r.χ).D=(φ.D+Ʈ.D+r.χ.D)=(Y+ƞ+r.D), hence proved.

## 5. Formal Security Analysis

In this section, the formal analysis for our proposed scheme is performed through the widely accepted ROR oracle model during the section, i.e., “4.3. Mutual Authentication and Secrete Key Management” between client and AP [28]. In Theorem 1, we proved that our designed scheme is safeguarded regarding derivations of the secret key (K=χ.ƞ and K=Q.φ) from both type of attacker, i.e., $A_{insd/out}=(A_{out}$, $A_{insd})$, which are shared between the client and AP. Furthermore, $A_{insd/out}$ has full access to the following queries:

*Execute Query:* With the help of this query, $A_{insd/out}$ can eavesdrop on all the transmitted messages between the client and AP.

*Corrupt Device Query:* With the help of this query, $A_{insd/out}$ can physically extract the parameters stored in the device that belongs to the client or AP.

*Reveal Query:* With the help of this query, $A_{insd/out}$ has access to a disclosed session key between the client and AP.

*Test Query:* With the help of this query, $A_{insd/out}$ can verify whether the generated session key is a random or real one.

**Theorem 1.**  *In this theorem, we prove that our scheme is a secret key that is secure from* $A_{insd/out}$ *, which can execute itself in a polynomial time (* $Pol_{tm}$ *). Suppose* $Q_{hqry}$ *,* $\left|Hash_{space}\right|$ *, and* $Adv_{A_{insd/out}}hecdlp$ *(* $Pol_{tm}$ *) denotes the hash query, space for hash value, and advantage of breaking the hardiness of* (hecdlp *) for* $A_{insd/out}$ *, respectively, then AdvAinsd/outhecdlp (Poltm)≤Qhqry2|Hashspace|+2AdvAinsd/outhecdlp (Poltm).*

**Proof.**  In this section, we made three games ($Game_{1}{}^{A_{insd/out}},Game_{2}{}^{A_{insd/out}},Game_{3}{}^{A_{insd/out}}$), and their explanations are followed. □

$Game_{1}{}^{A_{insd/out}}:$ By using this game, $A_{insd/out}$ can launch an actual attack on the proposed scheme and guess a random bit ($rdm_{bits}$), so we can obtain the following equation:

(1) $$Adv_{A_{insd/out}}hecdlp\;\left(Pol_{tm}\right)=\left|2Adv_{A_{insd/out,Game_{1}{}^{A_{insd/out}}}}proposed\;scheme\;\left(Pol_{tm}\right)-1\right|$$

$Game_{2}{}^{A_{insd/out}}:$ By using the execute query in this game, $A_{insd/out}$ can eavesdrop all the transmitted messages ((Q,r, S), (C)). Then, the attacker $A_{insd/out}$ can try to make the secret shared key (K=χ.ƞ and K=Q.φ). Furthermore, $A_{insd/out}$ needs to execute Reveal Query and Test Query to check whether the newly computed secret key is original or fake. Suppose their available outsider attacker ($A_{out}$) who is trying to generate K=χ.ƞ and decrypt (C). Suppose in our proposed scheme, $A_{out}$ has no access to the master secret key (𝓀) and has the capacity to replace the public key of the user. Therefore, in the proposed scheme, $A_{out}$ can extract the original value of the secret key by utilizing K=χ.ƞ and K=Q.φ; here, $A_{out}$ failed because, in these two equations, χ and φ are not known to them and also equals to find the solution for hyper elliptic curve discrete logarithm problem (hecdlp). Suppose their available insider attacker ($A_{insd}$) is trying to generate K=χ.ƞ and decrypt (C). Suppose in our proposed scheme, $A_{insd}$ has access to the master secret key (𝓀) and does not have the capacity to replace the public key of the user. Therefore, in the proposed scheme, $A_{insd}$ can extract the original value of the secret key by utilizing K=χ.ƞ and K=Q.φ; here, $A_{insd}$ failed because in these two equations χ and φ are not known to them and also equals to find the solution for hyper elliptic curve discrete logarithm problem. Thus, we can obtain the following equation.

(2) $$Adv_{A_{insd/out,Game2^{A_{insd/out}}}}proposed\;scheme=Adv_{A_{insd/out,Game1^{A_{insd/out}}}}proposed\;scheme$$

$Game_{3}{}^{A_{insd/out}}:$ By using the Corrupt Device Query, in this game $A_{insd/out}$ can derive the session key (K=χ.ƞ and K=Q.φ) by computing a hard problem such as hecdlp. The session key can be revealed in two ways, as follows: (1) Suppose in our proposed scheme, $A_{out}$ has no access to the master secret key (𝓀) and has the capacity to replace the public key of the user. Therefore, in the proposed scheme, $A_{out}$ can extract the original value of the secret key by utilizing K=χ.ƞ and K=Q.φ; here, $A_{out}$ failed because in these two equations χ and φ are not known to them and also equals to find the solution for hyper elliptic curve discrete logarithm problem (hecdlp). (2) Suppose their available insider attacker ($A_{insd}$) who is trying to generate K=χ.ƞ and decrypt (C). Suppose in our proposed scheme, $A_{insd}$ has access to the master secret key (𝓀) and does not have the capacity to replace the public key of the user. Therefore, in the proposed scheme, $A_{insd}$ can extract the original value of the secret key by utilizing K=χ.ƞ and K=Q.φ; here, $A_{insd}$ failed because in these two equations, χ and φ are not known to them and also equals to find the solution for hyper elliptic curve discrete logarithm problem. Moreover, the other credentials are protected through a hash function that is r= $H_{a}{}^{3}\left(\;Q.S,T_{limit},ƕ,Client_{PID}\right)$, so it is not possible for an attacker to recover these credentials because of the irreversible property of the hash function. Therefore, we can obtain the following equation:

(3) |AdvAinsdout,Game2Ainsdoutproposed scheme−AdvAinsdout,Game3Ainsdoutproposed scheme|          ≤Qhqry22|Hashspace|+AdvAinsd/outhecdlp (Poltm)

It is important to note that $A_{insd/out}$ is the only one who asks the queries; therefore, $A_{insd/out}$ must predict bits properly to win the game $Game_{3}{}^{A_{insd/out}}$. Therefore, we can obtain the following equation.

(4) $$Adv_{A_{insd/out,Game2^{A_{insd/out}}}}proposed\;scheme=\frac{1}{2}.\;$$

From Equation (1), we can obtain the following result.

(5) $$\begin{array}{c} \frac{1}{2}Adv_{A_{insd/out}}hecdlp\;\left(Pol_{tm}\right)=\\ \left|2Adv_{A_{insd/out,Game_{1}{}^{A_{insd/out}}}}proposed\;scheme\;\left(Pol_{tm}\right)-\frac{1}{2}\right| \end{array}$$

Then, by using Equations (2)–(4) with the help of triangular inequality, we can make the following results from Equation (5).

(6) 12AdvAinsd/outhecdlp (Poltm)=|AdvAinsdout,Game1Ainsdoutproposed scheme−AdvAinsdout,Game3Ainsdoutproposed scheme |=AdvAinsdout,Game2Ainsdoutproposed scheme−AdvAinsdout,Game3Ainsdoutproposed scheme |≤Qhqry22|Hashspace|+AdvAinsd/outhecdlp (Poltm)

By multiplying 2 by both sides of Equation (6), we can obtain the following result:

(7) AdvAinsd/outhecdlp (Poltm)≤Qhqry2|Hashspace|+2AdvAinsd/outhecdlp (Poltm).

## 6. Informal Security Analysis

The security analysis of the new scheme is based on the hard problem called hyper elliptic curve discrete logarithm problem, in which both types of attacker ($A_{out}$ and $A_{insd}$) trying to extract the unknown value, such as A from B=A.D. We consider two types of attacker, $A_{out}$ and $A_{insd}$; furthermore, $A_{out}$ is an outsider attacker who can try to steal information or destroy the forge ability and modify the medical data without having access to the master secret in a Dolev–Yao model channel. The $A_{insd}$ is the insider attacker who can try to steal information or destroy the forge ability and modify the medical data with access to master secret in a Dolev–Yao model channel. Hence, in the following sub phases, we illustrate the security analysis of our proposed scheme on the basis of a hyper elliptic curve discrete logarithm problem.

### 6.1. Confidentiality against $A_{out}$

Suppose there is an available outsider attacker ($A_{out}$) who is trying to generate K=χ.ƞ and decrypt (C). Suppose, in our proposed scheme, $A_{out}$ has no access to the master secret key (𝓀) and has the capacity to replace the public key of the user. Therefore, in the proposed scheme, $A_{out}$ can extract the original value of the secret key by utilizing K=χ.ƞ and K=Q.φ; here, $A_{out}$ failed because, in these two equations, χ and φ are not known to them and also equals to find the solution for hyper elliptic curve discrete logarithm problem (hecdlp).

### 6.2. Confidentiality against $A_{insd}$

Suppose their available insider attacker ($A_{insd}$) who is trying to generate K=χ.ƞ and decrypt (C). Suppose in our proposed scheme, $A_{insd}$ has access to the master secret key (𝓀) and does not have the capacity to replace the public key of the user. Therefore, in the proposed scheme, $A_{insd}$ can extract the original value of the secret key by utilizing K=χ.ƞ and K=Q.φ; here, $A_{insd}$ failed because in these two equations χ and φ are not known to them and also equals to find the solution for hyper elliptic curve discrete logarithm problem.

### 6.3. Unforgeability against $A_{out}$

Suppose their available outsider attacker ($A_{out}$) who is trying to generate S=φ+Ʈ+r.χ with the intention of making a forged signature. Suppose in our proposed scheme, $A_{out}$ has no access to the master secret key (𝓀) and has the capacity to replace the public key of the user. Therefore, in the proposed scheme, $A_{out}$ can extract the original value of S by utilizing S=φ+Ʈ+r.χ; here, $A_{out}$ failed because, in this equation, χ, φ, and Ʈ are not known to them and also equals to find the solution for hyper elliptic curve discrete logarithm problem three times.

### 6.4. Unforgeability against $A_{insd}$

Suppose their available insider attacker ($A_{insd}$) who is trying to generate S=φ+Ʈ+r.χ with the intention of making a forged signature. Suppose in our proposed scheme, $A_{insd}$ has access to a master secret key (𝓀) and does not have the capacity to replace the public key of the user. Therefore, in the proposed scheme, $A_{insd}$ can extract the original value of S by utilizing S=φ+Ʈ+r.χ; here, $A_{insd}$ failed because, in this equation, χ, φ, and Ʈ are not known to him and also equals to find the solution for hyper elliptic curve discrete logarithm problem three times.

### 6.5. Anonymity

In the proposed scheme, the client send (Q,r, S) to AP through an open network, where S=φ+Ʈ+r.χ, Q=χ.D, and r= $H_{a}{}^{3}\left(\;Q.S,T_{limit},ƕ,Client_{PID}\right)$. In this triple (Q,r, S), the client does not use any of its own or AP real identity, so we can say that our proposed scheme intelligently provides anonymity property.

### 6.6. Mutual Authentication

In the proposed scheme, the client can generate a signature S=φ+Ʈ+r.χ, and send (Q,r, S) to AP. When AP receives the triple (Q,r, S) it then performs the following step for the verification of the signature received from AP and generation of the secret key. It computes S.D=Y+ƞ+r.Q if it is qualified, then the client mutually authenticates with AP.

### 6.7. Modification Attack

In the proposed scheme, $\;A_{out}$ and $A_{insd}$ cannot modify the ciphertext because it is protected through a secret key K=χ.ƞ, so they can extract the original value of the secret key by utilizing K=χ.ƞ and K=Q.φ; here, $A_{insd}$ and $A_{out}$ failed because, in these two equations, χ and φ are not known to them and also equals to find the solution for hyper elliptic curve discrete logarithm problem.

### 6.8. Session Key Establishment

In the proposed scheme, A client can select χ randomly from the finite group of hyper elliptic curve and compute =χ.D, K=χ.ƞ, r= $H_{a}{}^{3}\left(\;Q.S,T_{limit},ƕ,Client_{PID}\right)$, S=φ+Ʈ+r.χ, and send (Q,r, S) to the client. When the client receives the triple (Q,r, S) then it performs the following step for the verification of the signature received from APR and generation of the secret key. It computes S.D=Y+ƞ+r.Q if it is qualified, then the client mutually authenticates with AP, then generate the secret key as K=Q.φ.

### 6.9. Impersonation Attack

In the proposed scheme, $A_{out}$ and $A_{insd}$ cannot generate the original signature as S=φ+Ʈ+r.χ. Suppose, in the proposed scheme, $A_{out}$ and $A_{insd}$ can extract the original value of S by utilizing S=φ+Ʈ+r.χ; here, $A_{out}$ and $A_{insd}$ failed because, in this equation χ, φ, and Ʈ are not known to him and also equals to find the solution for the hyper elliptic curve discrete logarithm problem three times.

## 7. Performance Evaluation

This section compares the proposed scheme to other relevant schemes in terms of computation and communication costs. The detailed comparative analysis regarding computation cost and communication between the proposed scheme and those of Wu et al. [22], He et al. [23], Ji et al. [24], and Xie et al. [25] are given in the following Section 7.1 and Section 7.2.

### 7.1. Computation Cost

**Figure 6:**
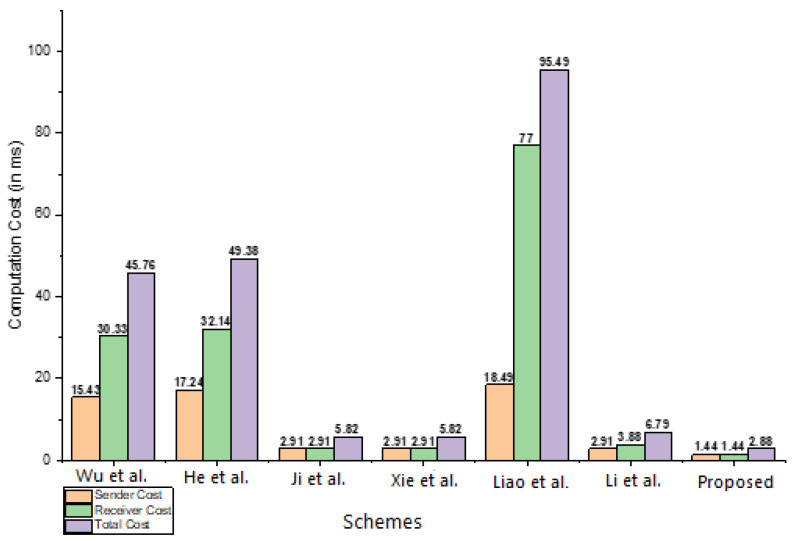
Computational cost comparison (in ms). Wu et al. [22], He et al. [23], Ji et al. [24], Xie et al. [25], Liao et al. [26], and Li et al. [27].

The proposed scheme is compared to the relevant schemes published by Wu et al. [22], He et al. [23], Ji et al. [24], Xie et al. [25], Liao et al. [26], and Li et al. [27] in this section. The comparison is made in terms of the cost of computation. The key findings from the computation cost comparison are summarized in Table 2. To assess the proposed scheme’s performance in terms of computation cost, we employed the Multi-precision Integer and Rational Arithmetic (MIRACL) C Library [29]. The library runs a large number of tests, up to 1000, on basic cryptographic operations. The simulations are run on a machine with a 2.0 GHz Intel Core i7-4510U CPU, 8 GB RAM, and Windows 7 [30]. Because of its smaller key size of 80 bits, the HEMUL is anticipated to take 0.48 milliseconds [31,32]. The comparisons are provided in Table 2, which reveals that the proposed scheme is substantially more cost-effective in terms of computation. The computational cost comparisons in milliseconds are also provided in Table 3, which is then illustrated in Figure 6 and clearly indicates that the proposed scheme is efficient by Wu et al. [22], He et al. [23], Ji et al. [24], Xie et al. [25], Liao et al. [26], and Li et al. [27].

### 7.2. Communication Cost

**Figure 7:**
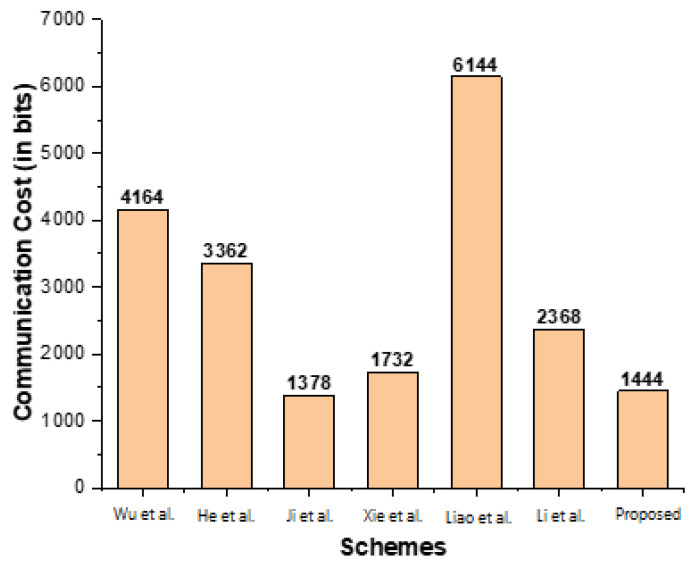
Communication cost comparison (in bits). Wu et al. [22], He et al. [23], Ji et al. [24], Xie et al. [25], Liao et al. [26], and Li et al. [27].

The proposed scheme is compared to the relevant schemes published by Wu et al. [22], He et al. [23], Ji et al. [24], Xie et al. [25], Liao et al. [26], and Li et al. [27] in this section. The comparison is made in terms of the communication cost. The key findings from the communication cost comparison are summarized in Table 4. The results show that the proposed scheme is better in communication cost than the existing schemes, which is also illustrated in Figure 7 and clearly indicates that the proposed scheme is efficient from Wu et al. [22], He et al. [23], Ji et al. [24], Xie et al. [25], Liao et al. [26], and Li et al. [27].

## 8. Conclusions

WBANs have recently received much attention as a result of recent technical developments in the fields of electronics, sensors, and wireless communication technologies, which allow patients to obtain preventative or proactive healthcare treatments from anywhere and at any time. Biomedical equipment, on the other hand, communicate regularly through an open wireless channel, making them vulnerable to a variety of cyber-attacks. In order to solve the security and privacy issues of WBAN, this article proposes an improved and efficient certificateless authentication scheme with a conditional privacy-preserving strategy. Hyperelliptic Curve Cryptography (HECC), a more sophisticated form of Elliptic Curve Cryptography, is used to build the proposed scheme (ECC). HECC offers the same degree of security while using a smaller key size, making it a more efficient solution than its alternatives. The proposed scheme, according to the comparative study, not only fulfills WBAN security and privacy criteria but also improves efficiency in terms of computation and communication costs.

In the future, we will propose a new heterogeneous authentication scheme in which the Key Generation Center can send the private key and partial private key through an open channel to the users without disclosing them to attackers.

## Figures and Tables

**Table 1:** Acronyms and symbols used in this paper.

No	Symbol/Acronym	Descriptions
1	WBAN	Represent Wireless Body Area Network
2	KGC	Used for Key Generation Center
3	$H_{a}{}^{1},H_{a}{}^{2},H_{a}{}^{3},H_{a}{}^{4}$	Represents four hash functions and their capability as it is irreversible
4	α	Used for the master public key of KGC
5	𝓀	Used for the master secret key of KGC
6	AP	Represents Application Provider
7	(ƞ,Y)	Represents the public key pair of Application Provider
8	(φ,Ʈ)	Represents the secret key pair of Application Provider
9	$Client_{PW}$	Represents the password for client
10	$Client_{RID}$	Represents the real identity for client
11	$Client_{PID}$	Represents the pseudo identity for client
12	⊕	Used for the encryption and decryption function
13	$E_{K}$	Encryption by utilizing the secret key K
14	$E_{K}$	Shared secret key which can be used for encryption and decryption of medical data
15	$T_{limit}$	It is used for to define the limit of time of session
16	$A_{out}$	Represents the attacking role of outsider attacker
17	$A_{insd}$	Represents the attacking role of insider attacker
18	MIRACL	Represents Multi-precision Integer and Rational Arithmetic
19	HEMUL	Used for Hyper elliptic curve divisor multiplication
20	$T_{\exp}$	Represents the time required for single exponentials
21	$T_{\mathrm{mp}}$	Represents the time required for single bilinear pairing multiplication
22	$T_{\mathrm{ecmp}}$	The time required for single elliptic curve multiplication
23	$T_{\mathrm{hecmp}}$	Time required for single hyper elliptic curve multiplication
24	$T_{p}$	Time required for single bilinear pairing operations
25	$b_{C}$	Represents the bits required for ciphertext
26	$b_{G}$	Represents the bits required for bilinear parameter
27	$b_{T}$	Rpresents the bits required for timestamp
28	$b_{q}$	Used for bits required for elliptic curve parameter
29	$b_{n}$	bits required for hyper elliptic curve parameter
30	$b_{h}$	It is used for bits required for hash value
31	HECC	Represents Hyperelliptic Curve Cryptography
32	PKG	Represents Private Key Generator
33	PKI	Represents Public Key Infrastructure
34	IBC	Used for Identity-Based Cryptography
35	ECG	Used to represent electrocardiogram
36	5G	Used to represent Fifth-Generation
37	ECC	Used to represent Elliptic Curve Cryptography

**Table 2:** Computational cost comparisons.

Schemes	Sender Cost	Receiver Cost	Total Cost
Wu et al. [22]	$2T_{\exp}$ + $3T_{\mathrm{mp}}$	$2T_{\exp}$ + $3T_{\mathrm{mp}}$ + $1T_{p}$	$4T_{\exp}$ + $4T_{\mathrm{mp}}$ + $1T_{p}$
He et al. [23]	$4T_{\mathrm{mp}}$	$1T_{p}$ + $4T_{\mathrm{mp}}$	$1T_{p}$ + $8T_{\mathrm{mp}}$
Ji et al. [24]	$3T_{\mathrm{ecmp}}$	$3T_{\mathrm{ecmp}}$	$6T_{\mathrm{ecmp}}$
Xie et al. [25]	$3T_{\mathrm{ecmp}}$	$3T_{\mathrm{ecmp}}$	$6T_{\mathrm{ecmp}}$
Liao et al. [26]	$4T_{\mathrm{mp}}$ + $T_{\exp}$	$2T_{\exp}$ + $5T_{p}$	$2T_{\exp}$ + $4T_{\mathrm{mp}}$ + $5T_{p}$
Li et al. [27]	$3T_{\mathrm{ecmp}}$	$4T_{\mathrm{ecmp}}$	$7T_{\mathrm{ecmp}}$
Proposed Scheme	$3T_{\mathrm{hecmp}}$	$3T_{\mathrm{hecmp}}$	$6T_{\mathrm{hecmp}}$

Note: $T_{\exp}=\mathrm{Time}\mathrm{required}\mathrm{for}\mathrm{single}\mathrm{exponentials}=1.25\;\mathrm{ms},$ $T_{\mathrm{mp}}=\mathrm{Time}\mathrm{required}\mathrm{for}\mathrm{single}\mathrm{bilinear}\mathrm{pairing}\mathrm{multiplication}=4.31\;\mathrm{ms},$ $T_{\mathrm{ecmp}}=\mathrm{Time}\mathrm{required}\mathrm{for}\mathrm{single}\mathrm{elliptic}\mathrm{curve}\mathrm{multiplication}=0.97,$ $T_{\mathrm{hecmp}}=\mathrm{Time}\mathrm{required}\mathrm{for}\mathrm{single}\mathrm{hyper}\mathrm{elliptic}\mathrm{curve}\mathrm{multiplication}=0.48,$ ${\mathrm{and}\;T}_{p}=\mathrm{Time}\mathrm{required}\mathrm{for}\mathrm{single}\mathrm{bilinear}\mathrm{pairing}\mathrm{operations}=14.90.$

**Table 3:** Computational cost comparisons in milliseconds.

Schemes	Sender Cost	Receiver Cost	Total Cost
Wu et al. [22]	2∗1.25+3∗4.31=15.43	2∗1.25 +3∗4.31 +1∗14.90=30.33	45.76
He et al. [23]	4∗4.31=17.24	1∗14.90+4∗4.31=32.14	49.38
Ji et al. [24]	3∗0.97=2.91	3∗0.97=2.91	5.82
Xie et al. [25]	3∗0.97=2.91	3∗0.97=2.91	5.82
Liao et al. [26]	4∗4.31+1.25=18.49	2∗1.25+5∗14.90=77	2∗1.25+4∗4.31+5∗14.90=95.49
Li et al. [27]	3∗0.97=2.91	4∗0.97=3.88	7∗0.97=6.79
Proposed Scheme	3∗0.48=1.44	3∗0.48=1.44	2.88

**Table 4:** Communication cost comparisons.

Schemes	Sender Cost	Total Cost in Bits
Wu et al. [22]	$2b_{C}$ $+\;2b_{G}+\;2b_{T}$	2∗1024 + 2∗1024+ 2∗34=4164
He et al. [23]	$1b_{C}$ $+\;2b_{G}+\;1b_{T}$ $+\;1b_{h}$	1∗1024 + 2∗1024+ 1∗34+ 1∗256=3362
Ji et al. [24]	$1b_{C}$ $+\;2b_{q}+\;1b_{T}$	1∗1024 + 2∗160+ 1∗34=1378
Xie et al. [25]	$1b_{C}$ $+\;4b_{q}+\;2b_{T}$	1∗1024 + 4∗160+ 2∗34=1732
Liao et al. [26]	$6b_{G}$	6∗1024=6144
Li et al. [27]	$2b_{C}$ $+\;2b_{q}$	2∗1024+2∗160=2368
Proposed Scheme	$1b_{C}$ $+2b_{h}$ $+\;1b_{h}$	1∗1024 + 2∗80+ 1∗256=1440

Note: ${\;b}_{C}=\mathrm{bits}\;\mathrm{required}\;\mathrm{for}\;\mathrm{ciphertext}=1024$, $b_{G}=\mathrm{bits}\;\mathrm{required}\;\mathrm{for}\;\mathrm{bilinear}\;\mathrm{parameter}=1024$, ${\;b}_{T}=\mathrm{bits}\;\mathrm{required}\;\mathrm{for}\;\mathrm{timestamp}=34$, $b_{q}=\mathrm{bits}\;\mathrm{required}\;\mathrm{for}\mathrm{elliptic}\;\mathrm{curve}\;\mathrm{parameter}=160$, ${\;b}_{n}=\mathrm{bits}\;\mathrm{required}\;\mathrm{for}\;\mathrm{hyper}\mathrm{elliptic}\;\mathrm{curve}\;\mathrm{parameter}=160,\;\mathrm{and}\;b_{h}=\mathrm{bits}\;\mathrm{required}\;\mathrm{for}\;\mathrm{hash}\;\mathrm{value}=256$.

## Data Availability

Not applicable.
